# Distribution of Cystic Fibrosis Transmembrane Conductance Regulator (CFTR) Mutations in a Cohort of Patients Residing in Palestine

**DOI:** 10.1371/journal.pone.0133890

**Published:** 2015-07-24

**Authors:** Issa Siryani, Mohamed Jama, Nisreen Rumman, Hiyam Marzouqa, Moein Kannan, Elaine Lyon, Musa Hindiyeh

**Affiliations:** 1 Caritas Baby Hospital, Bethlehem, Palestine; 2 Bethlehem University, Bethlehem, Palestine; 3 Palestinian Forum for Medical Research (PFMR), Ramallah, Palestine; 4 ARUP Institute for Clinical and Experimental Pathology, Salt Lake City, UT, United States of America; 5 Bethlehem University, Bethlehem, Palestine; 6 Department of Pathology, University of Utah, Salt Lake City, UT, United States of America; Heidelberg University, GERMANY

## Abstract

Cystic fibrosis (CF) is an autosomal recessive inherited life-threatening disorder that causes severe damage to the lungs and the digestive system. In Palestine, mutations in the Cystic Fibrosis Transmembrane Conductance Regulator gene (CFTR) that contributes to the clinical presentation of CF are ill defined. A cohort of thirty three clinically diagnosed CF patients from twenty one different Palestinian families residing in the central and southern part of Palestine were incorporated in this study. Sweat chloride testing was performed using the Sweat Chek Conductivity Analyzer (ELITECH Group, France) to confirm the clinical diagnosis of CF. In addition, nucleic acid from the patients’ blood samples was extracted and the CFTR mutation profiles were assessed by direct sequencing of the CFTR 27 exons and the intron-exon boundaries. For patient’s DNA samples where no homozygous or two heterozygous CFTR mutations were identified by exon sequencing, DNA samples were tested for deletions or duplications using SALSA MLPA probemix P091-D1 CFTR assay. Sweat chloride testing confirmed the clinical diagnosis of CF in those patients. All patients had NaCl conductivity >60mmol/l. In addition, nine different CFTR mutations were identified in all 21 different families evaluated. These mutations were c.1393-1G>A, F508del, W1282X, G85E, c.313delA, N1303K, deletion exons 17a-17b-18, deletion exons 17a-17b and Q1100P. c.1393-1G>A was shown to be the most frequent occurring mutation among tested families. We have profiled the underling mutations in the CFTR gene of a cohort of 21 different families affected by CF. Unlike other studies from the Arab countries where F508del was reported to be the most common mutation, in southern/central Palestine, the c.1393-1G>A appeared to be the most common. Further studies are needed per sample size and geographic distribution to account for other possible CFTR genetic alterations and their frequencies. Genotype/phenotype assessments are also recommended and finally carrier frequency should be ascertained.

## Introduction

CF is an inherited life-threatening disorder that causes severe damage to the lungs and the digestive system [1]. CF is caused by mutations in the gene that encodes the CFTR protein which is present on the epithelial and blood cells [1]. CFTR functions as a chloride channel in addition to other regulatory roles which includes inhibition of sodium transport through the epithelial sodium channel, regulation of intracellular vesicle transport, regulation of the outwardly rectifying chloride channel, regulation of ATP channels, and inhibition of endogenous calcium-activated chloride channels [1].

Analysis of sweat chloride is a laboratory “Gold Standard” for the diagnosis of CF [2]. The cut-off chloride ions value of 60mmol/L is considered diagnostic of CF, however in some patients chloride concentration might be less than 60 mmol/L but other clinical signs would also be present [3, 4].

CF is most common in European and European-derived populations [5]. The prevalence of CF is highest in Caucasians and Ashkenazi Jews; where one child in every 2,500 and 2,300 births is affected, respectively [6, 7]. Nevertheless, CF is present in other populations such as Native Americans (1 in 10,900), African Americans (1 in 15,000), and Asians (1 in 35,000) [7]. Although previously considered rare in those of non-Caucasian descent, CF has now been found in all ethnic groups, with an estimated prevalence of 1 in 10,000 births among Arabs and with affected individuals from India, Iran, South East Asia and Turkey [8].

More than 1800 CFTR mutations have been identified to date, with variable frequencies depending on the geographic and ethnic background [5]. The CF mutations present in the Palestinian population residing in Palestine are ill defined. Few studies from different countries reported the mutations present in the Palestinian patients they treated in those countries of which F508del, N1303K, W1282X, 3120+1Kbdel8.6Kb and G85E were the most common [9, 10]. In this study we evaluated the CFTR mutations in 33 clinically diagnosed CF Palestinian patients from 21 different families residing in the central and southern parts of Palestine.

## Materials and Methods

### Ethical Consideration

This study was approved by Caritas Baby Hospital (CBH) Medical Research Committee/Ethical Review Board (approval number: MRC-04). Written informed consents were obtained from the parents (father or mother) of the children involved in this study. All signed informed consent forms were deposited in the patient’s hospital medical chart.

### Patient Population

From September 2011 until November 2012, CBH personnel conducted a study to determine the CFTR mutations in a Palestinian cohort affected with CF. A total of thirty three patients from twenty one different families residing in the central and southern part of Palestine with the exception to Gaza district were incorporated in this study (Table 1). The majority of the families 17 (81%) came from Hebron district, followed by 3 (14.2%) from Bethlehem district and 1 (4.8%) from Ramallah district. Records of the patient’s family history and ancestry did not demonstrate that families with the same CFTR mutations were related or demonstrated any kinship. Haplotyping analysis (not performed in this study) of the recurrently occurring CFTR mutations may show that each mutation may occur on a common haplotype and thus may suggest a shared ancestry with true founder effect. It is also true that the shared CFTR mutation appeared only in few Palestinian families also may suggest that these families may be distantly related, rather than possessing a true founder mutation. The male to female ratio was 1:1.2 and the patients’ age ranged from 0.5–30 years.

**Table 1:** CFTR mutations distribution, sweat chloride testing results, clinical presentations and Palestinian patients’ demographics.

Code	Family	Members	Age(Years)	District	Result /Mutations	Sweat Conductivity Equivalent NaCl (mmol/L)	Pancreaticassessment	Sputum Culture Results	BMI
001	Pal-1	Daughter-1	5	Hebron	c.1393-1G>A	125	PI	*P*. *aeruginosa*	15.2
002	Pal-1	Daughter-2	11	Hebron	c.1393-1G>A	110	PI	*P*. *aeruginosa*	16.1
027	Pal-1/Cos	Daughter-1	7	Hebron	c.1393-1G>A	125	PI	*P*. *aeruginosa*	14.0
005	Pal-2	Daughter-1	5	Hebron	F508del	108	PI	*P*. *aeruginosa*	14.3
011	Pal-3	Son-1	25	Bethlehem	Deletion exons 17a-17b	137	PI	*P*. *aeruginosa*	18.4
013	Pal-4	Son-1	16	Hebron	W1282X	104	PI	*P*. *aeruginosa*	18.6
014	Pal-4	Daughter-1	5	Hebron	W1282X	119	PI	*P*. *aeruginosa*	16.1
015	Pal-4	Daughter-2	8	Hebron	W1282X	92	PI	*P*. *aeruginosa*	13.4
021	Pal-5	Son-1	14	Hebron	c.1393-1G>A	135	PI	NA	16.9
030	Pal-6	Daughter-1	2	Hebron	Het (c.1393-1G>A)Het (W1282X)	103	PI	*P*. *aeruginosa*	15.0
040	Pal-6/Cos	Son-1	11	Hebron	c.1393-1G>A	108	PI	*P*. *aeruginosa*	15.7
035	Pal-7	Son-1	6	Hebron	F508del	130	PI	Negative	14.0
036	Pa1-7	Daughter-1	10	Hebron	F508del	132	PI	Negative	15.7
038	Pal-8	Daughter-1	8	Hebron	Het (F508del)Deletion Exons 17a-17b-18	110	PI	*P*. *aeruginosa*	17.8
050	Pal-9	Daughter-1	14	Hebron	N1303K	132	PI	*P*. *aeruginosa*	12.6
058	Pal-10	Son-1	10	Hebron	Het (F508del)Deletion Exons 17a-17b-18	111	PI	*P*. *aeruginosa*	12.7
070	Pal-11	Daughter-1	4	Hebron	W1282X	101	PI	*P*. *aeruginosa* and MRSA	15.5
117	Pal-11/Cos	Daughter	0.5	Hebron	W1282X	120	PI	*P*. *aeruginosa*	13.3
072	Pa1-12	Daughter-1	5	Hebron	G85E	102	PI	*P*. *aeruginosa*	14.2
073	Pal-12	Daughter-2	7	Hebron	G85E	115	PI	*P*. *aeruginosa*	15.3
074	Pal-12/Cos	Daughter-1	11	Hebron	G85E	129	PI	*P*. *aeruginosa* and MRSA	16.2
079	Pal-13	Son-1	4	Hebron	444DelA	116	PI	MRSA	16.2
080	Pal-13	Son-2	7	Hebron	444DelA	101	PI	Negative	15.2
081	Pal-13	Son-3	1	Hebron	444DelA	90	PI	Negative	9.7
091	Pal-14	Son-1	7	Hebron	c.1393-1G>A	117	PI	Negative	15.2
093	Pal-15	Son-1	1	Hebron	F508del	117	PI	*P*. *aeruginosa*	15.2
099	Pal-16	Daughter-1	1	Hebron	W1282X	124	PI	*P*. *aeruginosa*	14.9
102	Pal-17	Son-1	30	Hebron	Het (G85E)Het (Q1100P)	130	PI	*P*. *aeruginosa*	20.1
105	Pal-18	Son-1	1	Ramallah	Deletion Exons 17a-17b-18	106	PI	Negative	13.6
107	Pal-19	Daughter-1	3	Bethlehem	c.1393-1G>A	115	PI	*P*. *aeruginosa*	12.7
111	Pal-20	Son-1	7	Bethlehem	c.1393-1G>A	148	PI	MRSA	14.5
112	Pal-20	Daughter-1	6	Bethlehem	c.1393-1G>A	113	PI	*P*. *aeruginosa* and MRSA	14.9
119	Pal-21	Daughter-1	6	Hebron	F508del	118	PI	*P*. *aeruginosa*	16.3

Pal = Palestine, Cos = cousin; BMI = Body Mass Index; PI = pancreatic insufficiency; Het = Heterozygous, MRSA = Methicillin Resistant *Staphylococcus aureus*; *P*. *aeruginosa = Pseudomonas aeruginosa*, NA = Not Available.

### Sweat Chloride Testing

The Sweat Chek^TM^ Conductivity Analyzer (ELITECH Group, France) was used to collect sweat samples from the patients and to determine the equivalent NaCl molarity [2, 11]. The Macroduct Sweat Collection System (Model 3700 SYS) kit was used and patients with NaCl molarity greater that 50mmol/L were considered suspicious for having CF and were referred to genetic testing [2, 4, 12].

### Blood Collection

Five to ten mL of patients’ peripheral blood were collected and placed into sterile EDTA tubes (MILIAN GENEVE COM-75). Samples were stored at 4–8°C pending analysis.

### DNA Extraction

Patients’ nucleic acid (NA) was extracted after blood treatment to lyse the Red Blood Cells (RBC). RBC lysis buffer (NH_4_Cl, NH_4_HCO_3_, EDTA 0.5M, *p*H: 7.4) 3 times the volume of the patients’ blood were mixed gently and kept refrigerated at 6–8°C for 45 minutes. Cell debris was then pelleted by centrifugation at 1,358 X g for 10 minutes. Supernatant was removed and the pellet was re-suspended in 5 ml RBC lysis buffer for 15 minutes at 6–8°C, and the remaining cell debris were then removed by centrifugation at 1,358 X g for 5 minutes. The pellet was then re-suspended in 500μl saline and mixed to homogenize the pellet. Patient’s genomic DNA was then extracted from 200μl using the High Pure Viral Nucleic Acid Kit (Roche Diagnostics, Switzerland) after following the manufacturer guidelines. Patients NA were eluted from the silica column using 200μl elution buffer.

### Polymerase Chain Reaction (PCR) and Sequencing

PCR amplification of the different 27 CFTR exons and the intron-exon boundaries were initially performed on the patients extracted nucleic acid as previously reported by Chou et al [13]. Amplified PCR products were sequenced bi-directionally using BigDye^®^ Terminator Cycle Sequencing kit version 1.1 (Applied Biosystems, Life Technologies Corporation, Carlsbad, CA) according to the manufacturer recommendations. Excess primers and dye terminators were then removed by centrifugation through a Centrisep spin column containing sephadex G-50 resin (GE Health Life Science, Pittsburgh, USA). Purified sequencing reaction products were then loaded up to Applied Biosystems 3730 DNA Analyzer (Applied Biosystems, Life Technologies Corporation, Carlsbad, CA).

### Sequence Results Analysis

Generated sequences were trimmed and aligned to GenBank CFTR reference sequence NG-016465, using Mutation Surveyor® software version 4.0.5 (SoftGenetics, LLC, State College, PA). Bioinformatics analysis was performed to detect any mutation or polymorphism in the patients’ CFTR exons and the intron-exon boundaries.

### Multiplex Ligation-Dependent Probe Amplification (MLPA)

DNA samples with no detectable pathogenic mutation as revealed by Sanger sequencing, were tested for deletions or duplications using SALSA MLPA probemix P091-D1 CFTR assay (MRC-Holland, Amsterdam, Netherlands). The assay was performed according to the manufacturer’s recommendation which targets all 27 exons and the promoter region of the CFTR gene [14]. All amplified products were loaded into ABI 3130 XL Genetic analyzer (Applied Biosystems, Foster City, USA) and peaks were sized using ROX 500 (Applied Biosystems, Foster City, USA). The relative peak size of the product from the probe recognition sequence was compared with the relative peak size of the product from a control. A 35 to 50% reduction indicated an exon deletion. The MLPA interpretation was facilitated by GeneMarker genotyping software (SoftGenetics, LLC, State College, PA).

## Results

### Sweat Chloride Testing

Sweat Chloride testing was performed on all the 33 patients from the 21 families. All patients had Sweat Conductivity Equivalent NaCl (mmol/L) above 50 mmol/L, range (90–148 mmol/L) (Table 1).

### Distribution of CFTR Mutations

Nine different CFTR mutations that were known to contribute to CF clinical presentations were detected in all 21 different families (Table 2). The CFTR mutations detected were c.1393-1G>A, F508del, W1282X, G85E, c.313delA, N1303K, deletion exons 17a-17b-18, deletion exons 17a-17b and Q1100P. Of the 21 families, 17 (81%) families had homozygous and 4 (19%) had compound heterozygous mutations (Table 2).

**Table 2:** Distribution of CFTR mutations in the Palestinian Families.

CFTR Mutations	Exon/Intron	# of Families	Frequency (%)
c.1393-1G>A / c.1393-1G>A	Intron 9	6	28.58
F508del / F508delA	Exon 10	4	19.05
W1282X / W1282X	Exon 20	3	14.29
F508del / Deletion exons 17a-17b-18	Exon 10 / Exons 17a-18	2	9.52
G85E / G85E	Exon 3	1	4.76
c.313delA / c.313delA	Exon 4	1	4.76
Deletion exons 17a-17b-18 / Deletion exons 17a-17b-18	Exons 17a-18	1	4.76
N1303K / N1303K	Exon 21	1	4.76
G85E / Q1100P	Exon 3 / Exon 17b	1	4.76
Deletion exons 17a-17b / Deletion exons 17a-17b	Exons 17a-17b	1	4.76

Intron 9 mutation, c.1393-1G>A, appeared to be the most common (28.6%), followed by F508del (19%) and W1282 (14.3%) in the cohort of Palestinian families evaluated. Interestingly, one Palestinian family (Table 1: Pal-6) had two different mutations in its family members, one family member was homozygous for the c.1393-1G>A mutation while his first cousin had a compound heterozygous mutations (c.1393-1G>A / W1282X). The other mutations listed in Table 2 appeared in single families, were mutations frequencies among the different families are shown. Most importantly, some mutations in particular, c.1393-1G>A and c.313delA were not reported before in the Palestinian population. Allele frequencies were not calculated in this study as the number of patients doesn’t reflect the whole Palestinian population as no samples were collected from Gaza and all the Northern districts of Palestine.

MLPA detected exons 17a-17b-18 deletions in the two patients, Pal-8/Daughter-1 and Pal-10/Son-1 (Table 1). In addition, MLPA did not detect any exons duplication in any of the patients tested. For Pat-3/Son-1, exons 17a-17b deletions were detected after repeated failures of PCR amplification.

### Clinical Presentation of CF Patients

Clinically, all the patients presented with pancreatic insufficiency requiring pancreatic enzyme replacement therapy (PERT) (Table 1). In addition, all patients had low Body Mass Index (BMI) ranging from 9.7 and 20.1 (Table 1). The majority of these patients had significant lung disease with reduced lung function and advanced bronchiectasis at a young age. On the microbiological level, three sputum cultures on all 32 CF patients revealed that 25 (75%) and 5 (16%) were positive for *P*. *aeruginosa* and MRSA, respectively. Interestingly, all the CF children with the c.313delA mutation were negative for *P*. *aeruginosa* in 3 times of repeated sputum cultures, but one of the three siblings was positive for Methicillin Resistant *Staphylococcus aureus* (MRSA). The life expectancy for CF patients has been increasing worldwide due to better CF care. However, in Palestine there is still lack of adequate resources for the treatment of such a chronic disease. Two patients in our cohort died at 8 and 17 years of age due to lung disease and a third patient died at age 5 years but the death was due to accidental trauma.

## Discussion

CF is considered the most common severe life-threatening autosomal recessive inherited disease of exocrine tissues characterized by the abnormal transport of ions and fluids across epithelial cell membranes [15–17]. Although CF is considered to be most common in Caucasians; its occurrence in Arabs is not common, and reports on CFTR gene mutations in Palestine are limited [18].

Unlike other countries where F508del is the most common mutation, this study showed that c.1393-1G>A (legacy name: 1525-1G>A) is the most occurring CFTR mutation among a cohort of Palestinians affected by CF despite the fact that this mutation is infrequent in the neighboring countries, and considered rare worldwide [19]. This splicing mutation was first described in 1993 in a CF patient of Indo-Iranian origin [20], and a subsequent study reported three Pakistani patients, two were homozygous and the third patient was heterozygous [21]. Recently, unlike the initial report by Wahab et al from Pakistan [21], Nikolic and co-workers stated that no homozygotes for this mutation have been reported so far [19]. Interestingly, this mutation was detected in six families out of the twenty one involved in our study in both homozygous (28.6%), and heterozygous (4.8%) forms.

As reported from several Arab countries, F508del was also found in CFTR patients in Palestine. Four families (19%) had this mutation in a homozygous form, while 9.5% families had a heterozygous form with Deletion Exons 17a-18. Previous reports showed the high incidence of F508del mutation in countries such as Israel, Jordan, Lebanon and areas of Arab Gulf [18]. In a cohort of 22 unrelated Lebanese patients with CF, F508del (34%) appeared to be the most common mutation followed by N1303K (27%), W1282X (7%), and S4X (7%). The CFTR mutation c.1393-1G>A did not appear to be present in any of the CF Arab patients evaluated [22].

In Israel, W1282X has been reported as the most common mutation in patients from Arab ethnic background [10]. This mutation appeared in three (14.3%) families, and has been reported in other Arab Countries. Other CFTR mutations have also been shown in our study with frequency of 4.76% (one family each). These mutations were G85E, N1303K, Q1100P, c.313delA, deletion Exons 17a-17b and 3120+1Kbdel8.6Kb (deletion in exons 17a, 17b, 18) [23]. Lerer et al reported the 3120+1Kbdel8.6Kb mutation in 1999 as a founder mutation in the Palestinian Arabs as our study showed three unrelated families having this mutation; two from the same region as heterozygous and one from another region as homozygous [23]. Interestingly, the rare c.313delA mutation which was noted in one Palestinian family (3 homozygous children) was also found in a single patient from an African descent [24]. A different distribution of the CFTR mutations in a cohort of 144 unrelated Jewish patients from different ethnic origins was reported by Quint et al [22]; where the F508del (35.6%) appeared to be the most common mutation followed by the W1282X (31.3%) mutation [22].

Identifying CFTR mutations in patients clinically presenting with CF symptoms is important not only to the patients and their relatives, but it also enables more reliable carrier detection in the population-screening program. At present, there is sufficient evidence to confirm that CF is present in Palestine and that the clinical presentations of the CF patients with the different type of CFTR mutations were similar to what reported worldwide. The newborn screening program using immunoreactive trypsinogen (IRT) has been used in many countries for early detection of CF [25]. Unfortunately, this screening test is not available in Palestine. Therefore, knowing the most common CFTR mutations in our population and developing a specific mutation screening kit will enable earlier and rapid diagnosis for our patients.

We call for the creation of a Palestinian CF diagnostic network with local and international collaboration. This network will not only help to collect reliable clinical and laboratory data in patients with CF symptoms, but it will guide the Palestinian physicians to better manage patients with CF. The Palestinian CFTR mutations detected in this Palestinian CF cohort were included to the CFTR2 data base.

### Ethical Considerations

This study was approved by Caritas Baby Hospital Medical Research Committee/Ethical Review Board (MRC-04). Written consents forms were obtained from the family members of all patients involved in the study.
